# Evaluation of a dimeric-cRGD peptide for targeted PET-CT imaging of peripheral angiogenesis in diabetic mice

**DOI:** 10.1038/s41598-018-23372-9

**Published:** 2018-03-29

**Authors:** Jamila Hedhli, Stephanie L. L. Slania, Agata Płoska, Andrzej Czerwinski, Christian J. Konopka, Marcin Wozniak, Maciej Banach, Iwona T. Dobrucki, Leszek Kalinowski, Lawrence W. Dobrucki

**Affiliations:** 1 Beckman Institute for Advanced Science and Technology, Urbana, IL USA; 2 Department of Bioengineering, University of Illinois at Urbana-Champaign, Urbana, IL USA; 3 Department of Medical Laboratory Diagnostics and Central Bank of Frozen Tissues & Genetic Specimens, Medical University of Gdansk, Gdansk, Poland; 4 Peptides International Inc, Louisville, KY USA; 5 Department of Hypertension, Medical University of Lodz, Lodz, Poland; 6 Biobanking and Biomolecular Resources Research Infrastructure Poland (BBMRI.PL), Gdansk, Poland

## Abstract

The *α*_*V*_ *β*_3_ integrin plays an important role in many physiological functions and pathological disorders. *α*_*V*_ *β*_3_ is minimally expressed in normal quiescent endothelial cells, but significantly upregulated during neovascularization. In this study, we evaluated a ^64^Cu-labeled dimeric cRGD tracer targeted at *α*_*V*_ *β*_3_ integrin and report its applicability to assess peripheral angiogenesis in diabetes mellitus (DM). We established a murine model of type-1 DM characterized by elevated glucose, glycated serum protein (GSP), and glycated hemoglobin A1c (HbA1c). We demonstrated that our imaging probe is specific to *α*_*V*_ *β*_3_ integrin under both normo- and hyperglycemic conditions. We found that the analysis of *in vivo* PET-CT images correlated well with gamma well counting (GWC). Both GWC and PET-CT imaging demonstrated increased uptake of ^64^Cu-NOTA-PEG4-cRGD_2_ in the ischemic hindlimb in contrast to non-ischemic control. GWC of the distal ischemic tissue from DM mice showed significantly lower probe accumulation than in non-DM mice. The immunofluorescence staining of the ischemic tissues showed a 3-fold reduction in CD31 and 4-fold reduction in the *α*_*V*_ *β*_3_ expression in DM vs. non-DM animals. In conclusion, we successfully demonstrated that diabetes-associated reductions in peripheral angiogenesis can be non-invasively detected with PET-CT imaging using targeted dimeric-cRGD probe.

## Introduction

Angiogenesis, the formation of new blood vessels from pre-existing microvasculature, represents an important and complex biological process. Blood vessels are the first organ to form in the embryo, and constitute the largest network in our body^1–3^. When the delicate balance between pro- and anti-angiogenic factors is disrupted, numerous physiological and pathological processes, including cancer and ischemic disease progression, can be affected^4,5^. Patients with diabetes mellitus (DM) in particular have been shown to have diminished capacity for neovascularization, which can lead to life-threatening complication in the heart and/or extremities, conditions known as coronary arterial disease (CAD) and peripheral arterial disease (PAD), respectively. So far, randomized clinical trials focused on the promotion of angiogenesis using local administration of growth factors like vascular endothelial growth factor (VEGF) or fibroblast growth factor (FGF) have shown no clear benefit in patients with CAD or PAD^5–7^. Careful interpretation of these results revealed that the unexpected failures could be attributed to a number of potential factors, including suboptimal delivery strategies, suboptimal duration of the therapy (which could lead to either insufficient growth of new vessels or excessive formation of nonfunctional vessels), and the myopic use of only a single growth factor at a time. Moreover, the evaluation of therapeutic angiogenesis in these trials was completed with relatively insensitive techniques focused on clinical endpoints like exercise tolerance, quality of life and survival, peripheral pressure measurements, and imaging of tissue perfusion.

More recently, focus within the scientific community has shifted towards the development of novel therapeutic strategies, such as genetic and stem cell-based approaches, as well as novel noninvasive imaging techniques to evaluate molecular events associated with angiogenesis^8–13^. It has been demonstrated that the extracellular matrix and integrins (including *α*_*V*_ *β*_3_ integrin a vitronectin receptor) are responsible for modulation of growth factor production in response to mechanical strain, and that they may play an integral role in the initiation of angiogenesis^14^. The physiological behavior of *α*_*V*_ *β*_3_, characterized by a very low expression in quiescent endothelium and upregulation in angiogenic cells, offer a tremendous advantage for the targeted imaging of angiogenesis. By imaging *α*_*V*_ *β*_3_ expression using radiolabeled probes, optimized for PET or SPECT, altered angiogenic activity may be diagnosed earlier and in a non-invasive manner, and the success of potential therapeutic strategies may be evaluated in almost real-time, which may lead to more individualized medical interventions and better patient outcomes.

Our group has previously evaluated a Technetium-99m labeled SPECT tracer (^99*m*^Tc-NC100692, maraciclatide)^15^ based on the arginine-glycine-aspartate (RGD) binding sequence for the imaging of *α*_*V*_ *β*_3_ integrin expression in various preclinical animal models of ischemia-induced angiogenesis^6,10^. This tracer, which showed a high *α*_*V*_ *β*_3_ affinity (1 nM) and a rapid and efficient renal clearance route, was successfully used to evaluate peripheral angiogenesis in a murine model of hindlimb ischemia, and myocardial angiogenesis in both mice and rats^6,10^. Building on this and work of others, the field has seen renewed interest in developing chemically modified cRGD-based tracers with the hope of optimizing their pharmacokinetics, biodistribution, and target affinity and specificity^16^. These efforts resulted in the development of several multimeric tracers, which are particularly useful in areas with multivalent binding sites and clusters of integrins^17–21^.

Initial work on dimeric cRGD compounds was performed in oncological research as a continuation of efforts focused on using *α*_*V*_ *β*_3_ integrin antagonists for the treatment of cancer^22–26^. Several groups showed that dimeric-cRGD molecules possess greater targeting capabilities and tumor uptake than their monomeric analogues^18^. Multivalency is a well established approach to increase the interaction of weakly interacting individual ligands with their respective receptors, and as a result, a number of constructs of greater cRGD multiplicity were also investigated^27^. It was found, however, that peptide multiplicities greater than two lead to only marginal enhancements in *in vitro* binding affinity, and these improvements did not directly lead to better imaging characteristics (in particular, highly multimeric probes have been plagued by non-specific rentention in organs like the liver, small intestines, and colon)^28^.

The goal of this investigation is to evaluate the feasibility of a novel multimeric cRGD tracer targeted at *α*_*V*_ *β*_3_ integrin for non-invasive PET imaging of peripheral angiogenesis in the onset of diabetes. The tracer was carefully characterized and used previously to evaluate myocardial angiogenesis in rats^17^. In the present study we demonstrate the feasibility of the probe for assessing angiogenesis in diabetic mice subjected to hindlimb ischemia. This imaging tracer was used to assess both temporal and spatial changes in local *α*_*V*_ *β*_3_ integrin expression in diabetic and non-diabetic animals subjected to surgical ligation of the right femoral artery in order to induce an angiogenic response. We detected a significant reduction in the *α*_*V*_ *β*_3_ activation in DM mice when compared to non-DM control mice. *En route* to this result, we established a number of other important findings relevant to both the murine model and the applicability of the probe for quantitatively assessing angiogenesis in diabetic mice. First, we show that the mouse model recapitulates several key markers of diabetes, including hyperglycemia, and enhanced GSP and HbA1c levels. Second, we demonstrate that a fluorescent analogue of the probe shows comparable specificity for *α*_*V*_ *β*_3_ an anti- *α*_*V*_ *β*_3_ antibody (LM609) when incubated under both normal and high glucose conditions. Third, we establish an optimal imaging protocol that allows for highly-detailed images. And finally, we validate the analysis of the PET-CT images and show a strong agreement with gamma well counting experiments on excised tissues, indicating that our probe can be used to accurately monitor *α*_*V*_ *β*_3_ expression in a non-invasive manner.

## Results

### Radiochemical purity and stability of the cRGD probes

The radiochemical purity of ^64^Cu-NOTA-PEG4-cRGD_2_, determined in both pH 7.4 PBS and blood plasma using C-18 RP-HPLC, was >95% up to 24 hrs after labeling^17^. The probe’s fluorescent analogue, FITC-PEG_4_-cRGD_2_, demonstrated similarly high stability in both media (data not shown).

### Animal model of peripheral angiogenesis in diabetes

#### Changes in blood biomarkers between 2 and 6 weeks after DM induction

**Figure 1:**
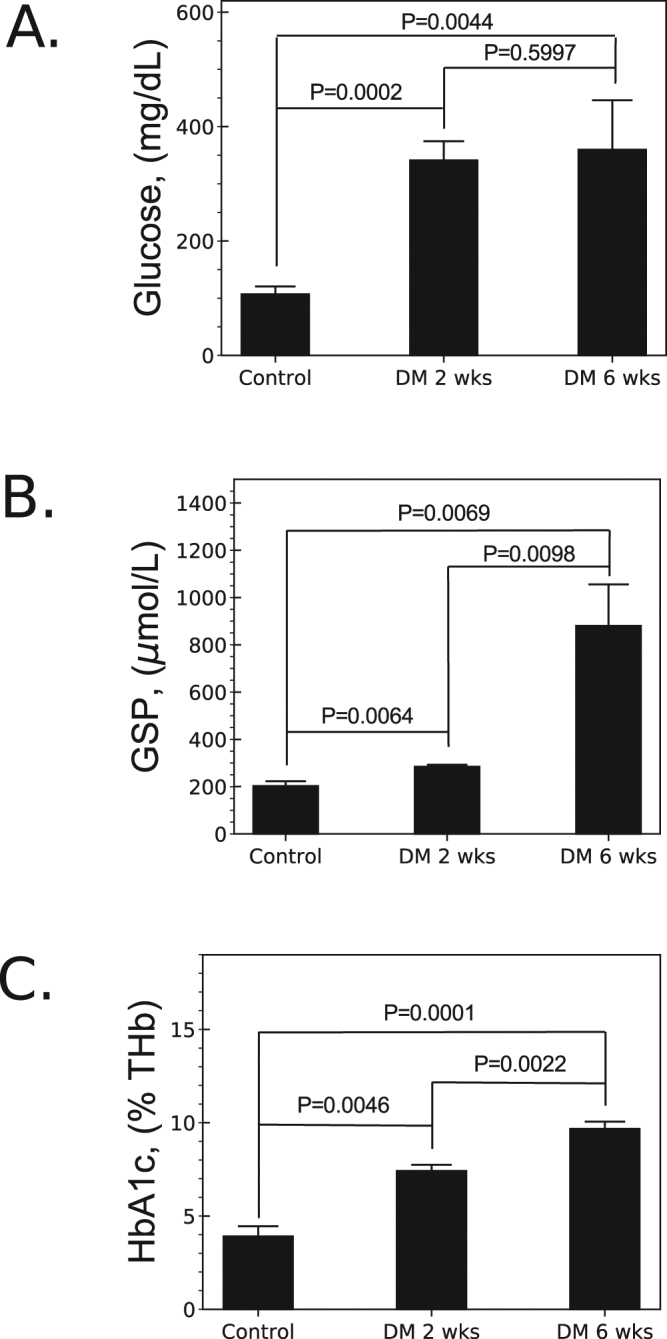
Characterization of diabetes-associated blood markers. (**A**) Glucose level was found to rise as soon as 2 weeks after induction of diabetes, but no further increase was found at the 6 week time point. (**B**) Glycated serum protein (GSP) showed an increase at 2 weeks, and surged dramatically by week 6. (**C**) Glycated hemoglobin A1c (HbA1c) expressed as a percent of total hemoglobin (THb) increased consistently over the 6 week time frame.

DM was introduced in a subset of mice through streptozotocin (STZ) treatment. After induction, we measured three key DM biomarkers: circulating blood glucose, GSP, and HbA1c blood levels. We detected a change in all three markers as early as two weeks after STZ administration. While we found no significant change in glucose level between weeks 2 and 6, we saw a significant surge in GSP and HbA1c levels (3 and 1.3-fold, respectively, see Fig. 1). These results indicate that many important diabetes-associated physiological changes can take up to 6 weeks to manifest in our murine model, and as such, we opted to conduct our *in vivo* experiments 6 weeks after STZ treatment.

#### Validation of the animal model of peripheral ischemia

**Figure 2:**
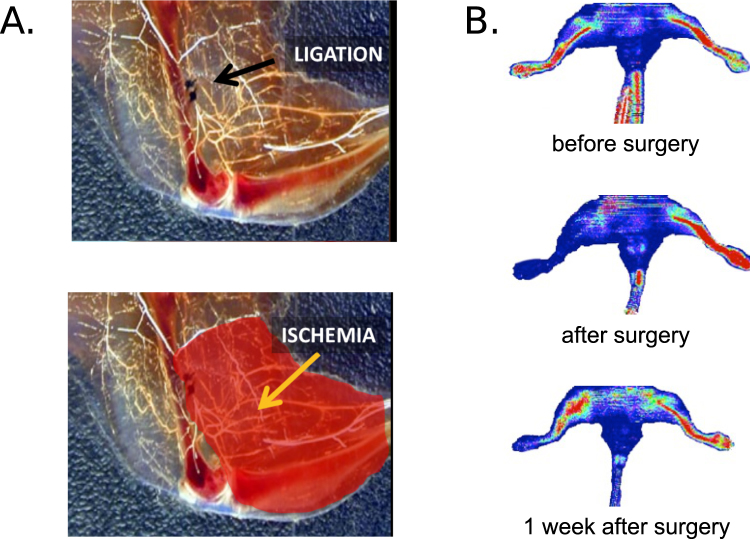
Surgical model of murine hindlimb ischemia. (**A**) Animals underwent surgical occlusion of the right femoral artery by placing two ligatures distal to profundus branch inducing unilateral hindlimb ischemia (bottom, yellow arrow). Placement of ligatures (top,black arrow) and vascular occlusion was visualized with the Microfil casting and tissue clearing technique. (**B**) The surgery resulted in an immediate decrease in perfusion in ischemic hindlimb with a partial recovery at 1 week after the surgery, as assessed using Laser Doppler flowmetry.

The right femoral arteries of the animals were surgically ligated in order to model peripheral vascular occlusion. This resulted in distal ischemia followed by the initiation of both the angiogenic and arteriogenic processes. Microfil casting and tissue clearing techniques were used in a subset of animals to visualize the location of the occlusion and the predicted ischemic area in relation to other anatomical structures (see Fig. 2). To verify the completeness of the surgical ligations, we performed Laser Doppler imaging of peripheral perfusion. Immediately after the surgery we observed dramatic reduction (>80%) in blood perfusion within the ischemic hindlimb, which was partially recovered at 1 week after the ligation (see Fig. 2). Based on this observation, and results published previously^17^, we used the 1 week time point to study the differences in peripheral angiogenesis in our DM and non-DM mice.

### Properties Of FITC-PEG_4_-cRGD_2_–*α*_*V*_*β*_3_ binding In a high-glucose microenviroment

**Figure 3:**
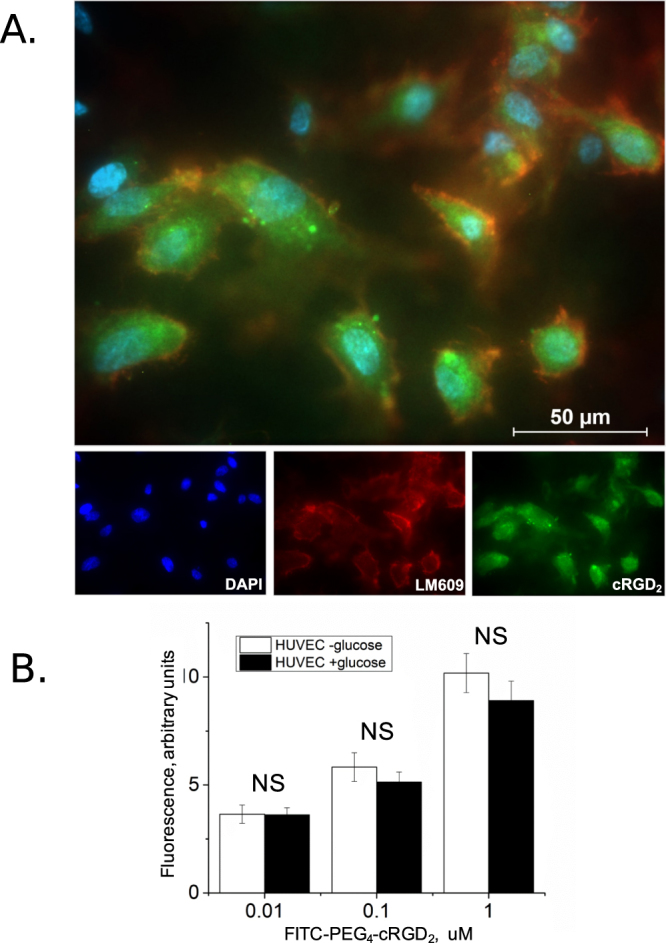
Colocalization of FITC-PEG_4_-cRGD_2_ and LM609 to human umbilical vein endothelial cells (HUVECs) expressing *α*_*V*_ *β*_3_ integrin. (**A**) Incubation of HUVECs with 1 *μ* M FITC-PEG_4_-cRGD_2_ (green) and phycoerythrin-conjugated LM609 (1:100, red). The high degree of overlap indicates specific binding of our probe to *α*_*V*_ *β*_3_. (**B**) HUVECs incubated for 24 hrs in low-glucose (5.5 mM) or high-glucose (14 mM) medium did not result in any significant differences in FITC-PEG_4_-cRGD_2_ uptake, as assessed by flow cytometry.

The specificity of the probe was assessed by co-incubating FITC-PEG_4_-cRGD_2_ and the commercially available phycoerythrin-labeled *α*_*V*_ *β*_3_ antibody, PE-LM609. For these experiments we used human umbilical vein endothelial cells (HUVECs), which are known to constitutively express *α*_*V*_ *β*_3_. As shown in Fig. 3 there is a strong colocalization between the fluorescein and phycoerythrin signals. This is in accordance with our previously published studies showing a high correlation between FITC-PEG_4_-cRGD_2_ and PE-LM609 fluorescence using single cell flow cytometry^17^.

Of particular importance to imaging *α*_*V*_ *β*_3_ expression in DM patients is to determine whether a high-glucose environment can affect the *α*_*V*_ *β*_3_ receptor’s binding properties. To exclude this possibility, we incubated HUVECs with the FITC-PEG_4_-cRGD_2_ in growth media supplemented with 14 mM glucose. Comparison with a control HUVECs cultured in normal growth media showed no significant difference in FITC-PEG_4_-cRGD_2_ uptake under elevated glucose conditions (see Fig. 3). This observation indicates that any differences in the uptake of the imaging probe that arise between diabetic and non-diabetic animals is likely due to changes in *α*_*V*_ *β*_3_ integrin expression/activation levels and not glycation-associated modification of the vitronectin receptor.

### Pharmacokinetics and biodistribution

**Figure 4:**
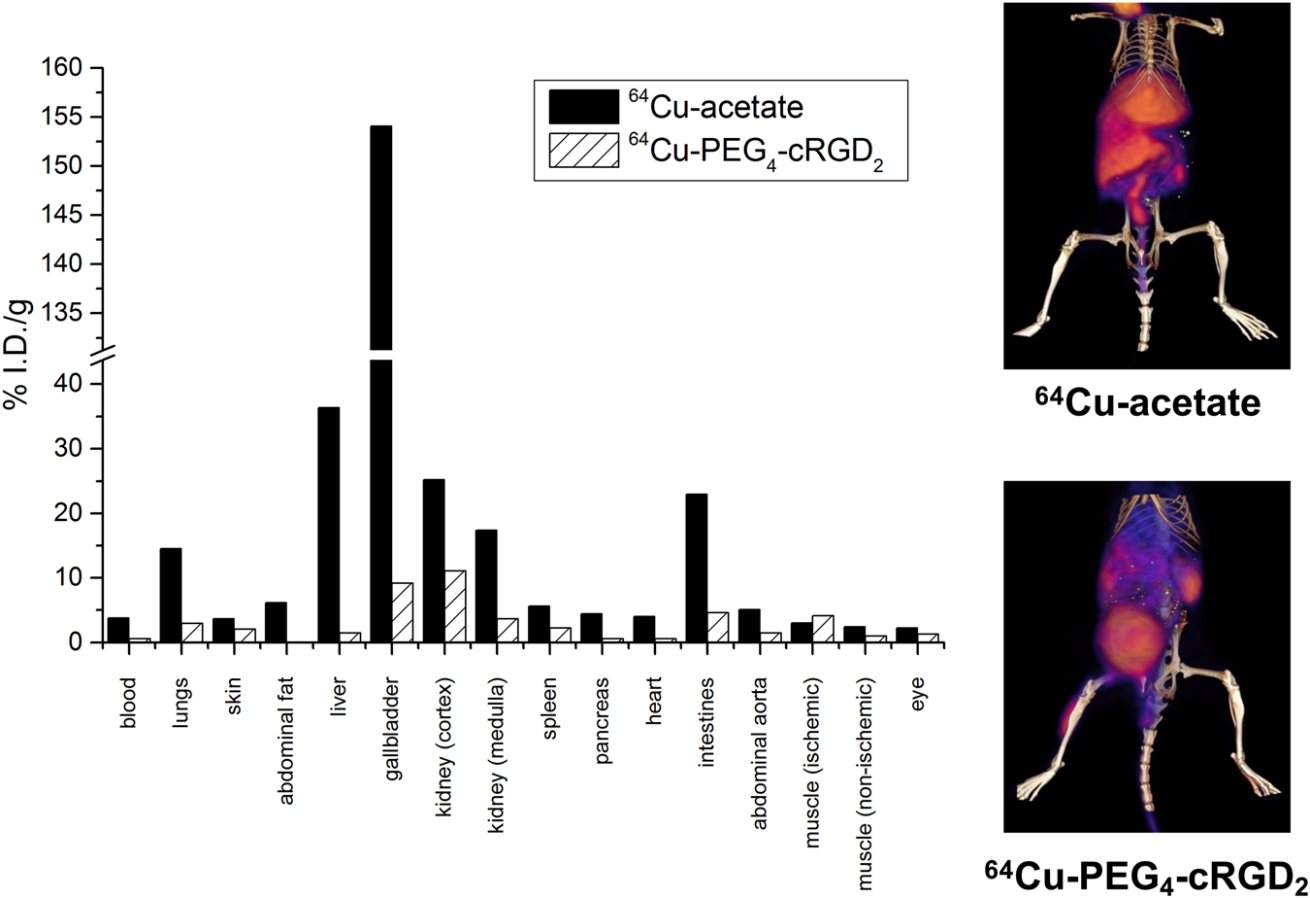
Organ-specific uptake (right, assessed using PET-CT) and biodistribution (left, assessed using gamma well counting) of ^64^Cu-NOTA-PEG4-cRGD_2_ and the non-targeted ^64^Cu-acetate one hour after jugular vein injection demonstrates a favorable biodistribution and optimal retention for targeted *in vivo* imaging of peripheral angiogenesis.

To demonstrate that the radiotracer exhibits favorable organ retention for the purpose of imaging peripheral angiogenesis, we performed comparative biodistribution studies using both targeted ^64^Cu-NOTA-PEG4-cRGD_2_ and the non-targeted ^64^Cu-acetate as control. Figure 4 shows the difference in the biodistribution profiles of both radiotracers. As expected, non-targeted ^64^Cu-acetate demonstrated increased uptake in all organs (including blood) with a very high uptake (>20%*I*.*D*./*g*) in the liver, gallbladder, kidneys, and intestines due to non-specific affinity to tissue proteins. In contrast, ^64^Cu-NOTA-PEG4-cRGD_2_ uptake was significant in the gallbladder, kidney cortex and intestines. In addition, PET imaging revealed that ^64^Cu-NOTA-PEG4-cRGD_2_ cleared rapidly from the blood (contributing to an overall low background) and was excreted predominantly by kidney filtration.

**Figure 5:**
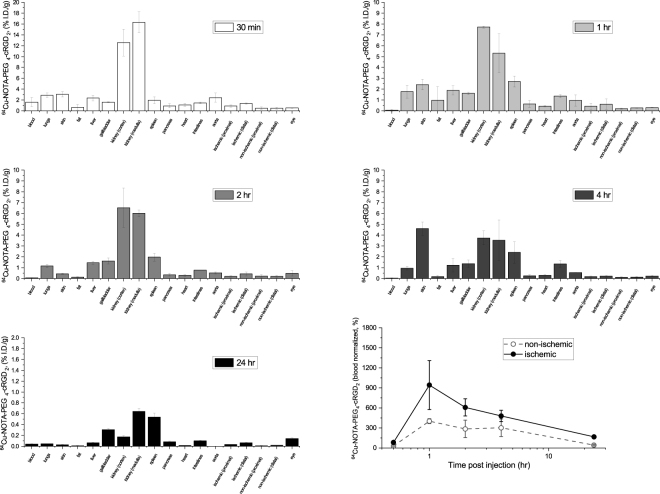
Biodistribution of ^64^Cu-NOTA-PEG4-cRGD_2_ at various time points after injection. At the 30 min time point, the probe was mainly in the bloodstream, while the one and two hour time points showed the greatest accumulation in the distal ischemic tissue. Later time points showed little accumulation in the ischemic limb, and largely resembled the non-ischemic tissue. These results indicate the optimal time point for imaging is between one and two hours after administration of the tracer.

We performed additional biodistribution studies of ^64^Cu-NOTA-PEG4-cRGD_2_ at a series of time points (30 min, 1 hr, 2 hr, 4 h and 24 hr after injection) to evaluate the clearance of the probe and establish the optimal post-injection timing for a PET-CT scan (see Fig. 5). We found that PET imaging at 1–2 hours post injection resulted in the optimal blood clearance and uptake within the ischemic muscle.

### PET-CT imaging analysis versus gamma well counting

**Figure 6:**
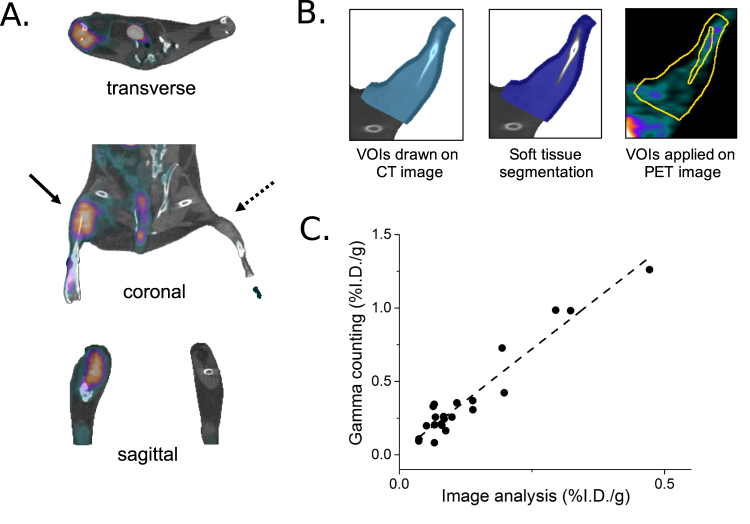
PET-CT imaging analysis. (**A**) Representative *in vivo* PET-CT images of peripheral angiogenesis 1 week after surgical ligation of the right femoral artery. One hour after intravascular injection of ^64^Cu-NOTA-PEG4-cRGD_2_ a significant uptake of the radiotracer (“hot spot”) was observed in the ischemic hindlimb. (**B**) For the image analysis, volumes-of-interests (VOIs) were drawn on CT images of ischemic and non-ischemic hindlimbs (left) followed by segmentation of hindlimb muscles (middle). These irregular VOIs were placed on PET images to calculate the radiotracer’s uptake (expressed in %I.D./g tissue, right). (**C**) Correlation between the two methods (PET-CT and gamma well counting) used to measure radiotracer uptake in mice hindlimbs showed a strong linear correlation (*R*^2^ = 0.9602, solid line).

Representative PET-CT images of peripheral angiogenesis acquired 1 week after surgical ligation are shown in Fig. 6. Confirmed by biodistribution studies, PET-CT images obtained 1 hr after intravenous injection of the radiotracer were of excellent quality and demonstrated “hot spots”–of increased ^64^Cu-NOTA-PEG4-cRGD_2_ uptake within the ischemic hindlimb–whereas the non-ischemic muscle showed no significant uptake. Moreover, VOI-based image analysis of PET-CT images (see Fig. 6) correlated well (R^2^ = 0.9602) with postmortem ^64^Cu-NOTA-PEG4-cRGD_2_ activities in the hindlimb muscle sections measured using gamma well counting immediately after PET-CT imaging (see Fig. 6).

### Diabetes-associated reductions in *α*_*V*_ *β*_3_ can be monitored non-invasively using ^64^Cu-NOTA-PEG4-cRGD_2_

**Figure 7:**
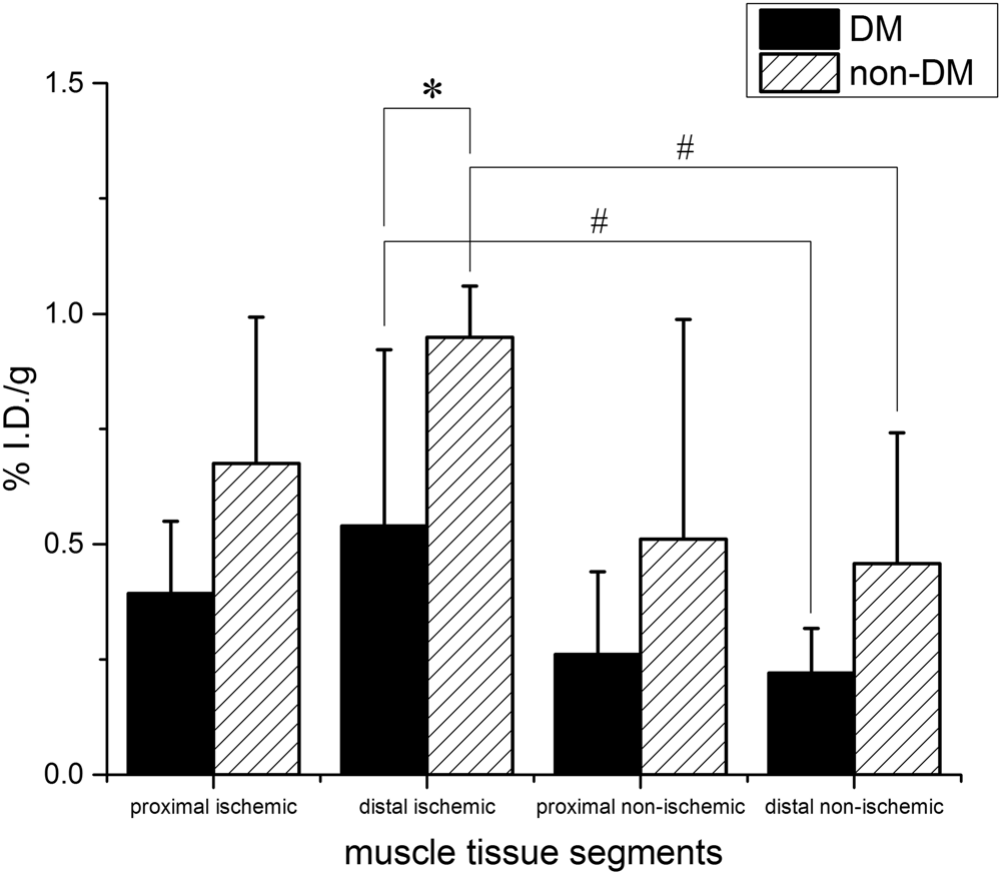
Analysis of ^64^Cu-NOTA-PEG4-cRGD_2_ retention in the hindlimbs of DM and non-DM mice 1 week after surgical ligation of the right femoral artery. Overall there was a significant (^#^*P* < 0.05) increase in ^64^Cu-NOTA-PEG4-cRGD_2_ retention in the distal segments of the ischemic relative to the non-ischemic hindlimbs, as well as a significant (*P < 0.05) decrease in the distal ischemic hindlimb of DM mice compared to non-DM controls.

Most importantly, we found a significant (*P* < 0.05) reduction of ^64^Cu-NOTA-PEG4-cRGD_2_ uptake in all muscle segments (distal and proximal) of diabetic animals when compared to the non-diabetic control segments (see Fig. 7). Quantitative analysis of PET-CT images confirmed this result, indicating that DM mice had significantly lower %I.D./g in their distal ischemic tissue than non-diabetic mice. PET imaging showed a significant increase in the retention of ^64^Cu-NOTA-PEG4-cRGD_2_ in the ischemic (relative to non-ischemic) hindlimbs, which demonstrates the probe’s specificity to areas of active angiogenesis and *α*_*V*_ *β*_3_ expression. Together, these findings indicate that molecular imaging with ^64^Cu-NOTA-PEG4-cRGD_2_ is suitable to quantitatively asses different angiogenic responses in DM and non-DM environments.

**Figure 8:**
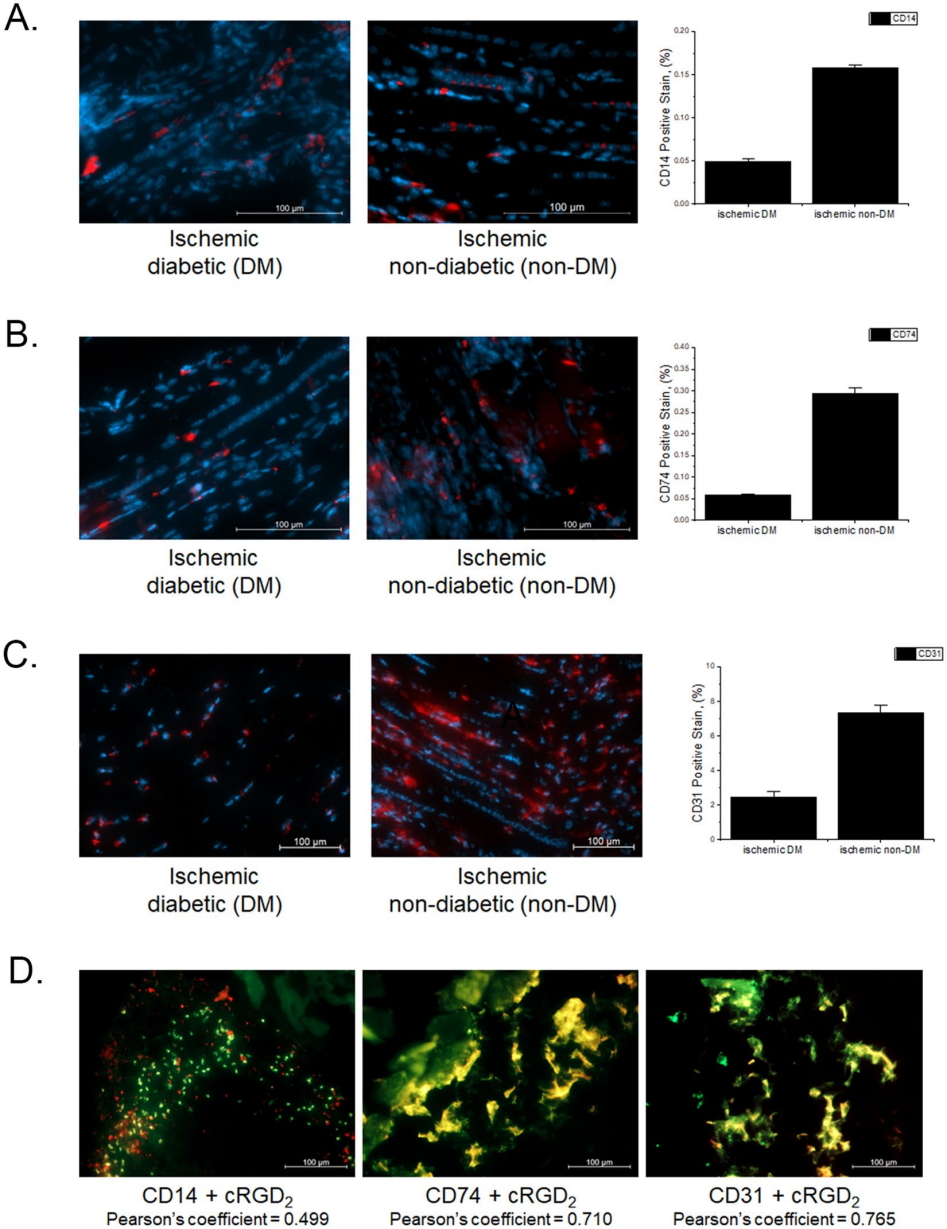
Representative cross sections taken from ischemic lower hindlimbs of both DM (left) and non-DM (right) mice 1 week after femoral occlusion. (**A**) Sections were stained with inflammation marker CD14 (red) and co-stained with DAPI (blue) to visualize nuclei. (**B**) Sections were stained with reperfusion marker CD74 (red) and co-stained with DAPI (blue) to visualize nuclei. (**C**) Sections were stained with endothelial marker CD31 (red) and co-stained with DAPI (blue) to visualize nuclei. (**D**) Sections were stained with CD14, or CD74, or CD31 (red) and fluorescent analogue FITC-PEG_4_-cRGD_2_. Fluorescence quantification showed a reduction in both capillary density (CD31 staining), inflammation (CD14), reperfusion (CD74), and FITC-PEG_4_-cRGD_2_ (*α*_*V*_ *β*_3_) retention in DM animals when compared with non-DM controls *P < 0.05. We found a strong correlation between CD31, CD74 and FITC-PEG_4_-cRGD_2_, and a modest degree of co-localization between CD14 and FITC-PEG_4_-cRGD_2_.

In order to investigate the physiological effects that are associated with *α*_*V*_ *β*_3_ expression in both DM and non-DM mice, we evaluated tissue samples collected from all animals. Both ischemic and non-ischemic distal hindlimb muscle sections were stained with FITC-PEG_4_-cRGD_2_, Cy-5 labeled endothelial cell marker (CD31), CD14 (a marker mostly associated with macrophages and dendritic cells), or CD74 (a cell surface receptor expressed by macrophages and endothelium cells which has been linked to reperfusion after vascular injury^29^). Representative immunofluorescence images taken from DM and non-DM mice are shown in Fig. 8A,B,C. We found CD31 positive endothelial cells within the capillaries and small arterioles of the non-diabetic ischemic hindlimb sections, which correlated with high *α*_*V*_ *β*_3_ expression. By comparison, the DM mice showed significant reduction in CD31 positive staining and FITC-PEG_4_-cRGD_2_ (3.5- and 3.8-fold respectively) in their ischemic muscle tissues, suggesting both a reduction in capillary density and decreased *α*_*V*_ *β*_3_ expression in DM animals relative to the non-DM controls. We found as well that DM mice showed a statistical significant reduction (3.2 and 4 fold) for both CD14 and CD74 respectively as shown in Fig. 8A,B. These results demonstrate the dynamic changes in the DM microenvironment. We performed additional histology studies to determine the extent of co-localization between the FITC-labeled probe and CD14 or CD74. The FITC-PEG_4_-cRGD_2_ showed a modest degree of co-localization (Pearson coefficient of 0.50) with the labeled CD14, but a much stronger correlation (Pearson coefficient of 0.71) with the labeled CD74 see Fig. 8D. This supports the idea that our probe is effectively targeting regions of active angiogenesis and vascular repair. As expected, the greatest degree of co-localization occurred with CD31 (correlation coefficient of 0.765), indicating that the probe targets endothelial cells much more strongly than macrophages, dendritic cells, or other cell types associated with the ischemic response. Finally, we also found that both CD14 and CD74 staining were significantly reduced in DM relative to non-DM ischemic tissues (by about 3.2 and 4.0 fold, respectively). This once again underscores the negative impact of DM on vascular regeneration.

## Discussion

The study presented here focuses on the feasibility of assessing diabetes-associated differences in peripheral angiogenesis using a ^64^Cu labeled dimeric cyclic RGD-based PET-CT probe targeted at *α*_*V*_ *β*_3_. The design of our probe benefitted from years of prior results; it is characterized by high chemical stability, a favorable biodistribution, and a focal retention that results in an excellent quality of obtained images. ^64^Cu- was chosen as a PET imaging isotope due to its relatively easy chemistry, the availability of strong chelators capable of providing a firm backbone for the radioactive label, and the potential for clinical translation due to its relatively long half-life (~12.7 hours). Furthermore, a fluorescent analogue of our targeted tracer (FITC-PEG_4_-cRGD_2_), was developed and successfully used for *in vitro* studies in cell and tissue samples. The availability of this type of dual-modality probe may allow for future clinical translation in the field of image-guided surgery or intraoperative microscopy.

The *α*_*V*_ *β*_3_-targeted probes used in this study have been characterized by our group in detail previously^17^. Briefly, in order to verify its specificity and cellular binding kinetics, we evaluated the colocalization of FITC-PEG_4_-cRGD_2_ with the *α*_*V*_ *β*_3_-specific antibody LM609 in cultured integrin-expressing endothelial cells (HUVECs, chosen because there are no mouse-specific antibodies for *α*_*V*_ *β*_3_). Importantly, because diabetic animals are known to have uncontrollably high circulating glucose levels (which can alter the endothelial microevironment, induce conformational changes in the vitronectin receptor, and possibly impact other receptor-ligand interactions), in the present work, we also investigated the binding affinity of FITC-PEG_4_-cRGD_2_ in a high-glucose environment. We observed no significant change across a range of probe concentrations spaning from 0.01 to 1 *μ* M. This indicates that the differences in the angiogenic response we observed between diabetic and non-diabetic mice were associated with physiological changes due to changes in *α*_*V*_ *β*_3_ expression and not the binding affinity of the probe. Intriguingly, we note a relative decrease in probe accumulation in the non-ischemic diabetic limbs relative to the non-diabetic limbs. Because the binding affinity of our probe is unaffected by a high-glucose environment, we attribute this to a decrease in *α*_*V*_ *β*_3_ expression in the diabetic animals.

Following the *in vitro* applications, we evaluated the specificity of the probe *in vivo*. The labeling protocol we use involves first dissolving ^64^Cu in acetate buffer, and then combining that with a solution of our unlabeled probe. It is therefore important to show that labeled acetate exhibits no obvious accumulation in ischemic tissue. We subjected animals to a surgical femoral artery ligation and injected them with either ^64^Cu-NOTA-PEG4-cRGD_2_ or the non-specific ^64^Cu-acetate. We found a greater accumulation of ^64^Cu-NOTA-PEG4-cRGD_2_ in the ischemic hindlimb relative to the non-ischemic hindlimb while ^64^Cu-acetate showed comparable accumulation in both limbs paralleled by strong uptake in other organs. This further verifies the suitability of the ^64^Cu-NOTA-PEG4-cRGD_2_ for *in vivo* targeted imaging of angiogenesis.

In order to establish that the animal model used in our investigations faithfully mimics diabetes-associated pheripheral ischemia in human patients, we measured a number of important diabetic markers in experimental animals. The animals were first treated with STZ for three days which resulted in significant increase of circulating blood glucose levels. In humans, this can caused changes in both the short term (altering the expression levels of several proteins) and long term (giving rise to glycosylated hemoglobin)^30,31^. Our animals showed similar clinical signs of short- and long-term effects, including enhanced GSP and HbA1c levels (taking about 6 weeks after STZ treatment to fully materialize). Moreover, these enhancements were found to correlate with the decrease in angiogenic response confirmed with PET-CT imaging. Taken together, these findings represent an important part of establishing the utility of our model for the study of diabetes-associated vascular complications.

We next sought to establish the optimal time point for PET-CT imaging after administration of the ^64^Cu-NOTA-PEG4-cRGD_2_ by performing biodistribution studies at various time points after injection of the radiotracer. We found a time interval between 1 and 2 hours to be optimal, demonstrating low blood activities and significant accumulation within the ischemic tissue. We found that the main excretion route was via the bladder, which is favorable for probes with translational applicability because it avoids accumulation within the digestive track, and poses relatively little risk to other organs.

We validated the results from the PET-CT imaging and quantitative image analysis by postmortem evaluation of the radioactivity of excised tissues using gamma well counting. Gamma well counting is traditionally considered a “gold-standard” technique that enables measurement the absolute values of radiotracer activity. It is immune to several limitations that image-based whole body analyses face, including the partial volume effect and tissue attenuation. Unfortunately, gamma well counting is highly invasive, the animals need to be euthanized and tissues samples collected postmortem. We found a strong positive correlation between the image-based analysis and gamma well counting results. As expected, uncorrected PET-CT image analysis tended to underestimate the magnitude of the absolute ^64^Cu-NOTA-PEG4-cRGD_2_ uptake (expressed as %I.D./g) which we attribute predominantly to partial volume errors (tissue attenuation is negligible in small animals), detector sensitivity and different energy ranges. This discrepancy could be potentially eliminated using partial volume correction techniques, normalization of detector efficiency and allowing comparable energy ranges.

The results of the PET-CT study strongly suggest reduced *α*_*V*_ *β*_3_ activity in the onset of diabetes, which negatively affects the angiogenic process. This observation is further verified by immunofluorescence staining in which diabetic tissues showed a significant reduction in the expression of CD31, CD14, and CD74 (markers of endothelial cells, macrophages, dendritic cells, and vascular repair) and retention of FITC-PEG_4_-cRGD_2_. This finding is in agreement with studies performed previously by our group using both SPECT and PET cRGD-based tracers, and by others using more invasive techniques^32^. Diabetes-induced impairment of collateral blood vessel formation has also been demonstrated in other preclinical animal models of diabetes^17,33^.

While the underlying causes are not completely understood, attenuated angiogenesis in diabetes has been linked to improper degradation of the basement membrane, to alterations in the delicate balance of growth factors and cytokines that regulate vascular stability, and to problems in signal transduction including VEGF dysregulation (as shown by reduced expression of VEGF mRNA and protein)^32^. Rivard *et al*. in particular, demonstrated that hindlimb ischemia created by ligation of the femoral artery was associated with a greater reduction in capillary formation and blood flow to the ischemic limb in diabetic transgenic mice (NOD) relative to non-diabetic (C57) wild type mice. In these studies, NOD mice showed a much lower rate of perfusion (ischemic limb to normal limb) 14 days following femoral artery ligation and a reduction in capillary density in ischemic hindlimb muscles at 35 days when compared with healthy littermates^32^.

Although others have investigated the effects of diabetes on neovascularization, most have relied on invasive assessment techniques with little applicability in the clinical setting. Due to its non-invasive nature, PET-CT imaging represents a powerful tool for studying the vascular complication of diabetes, in the laboratory, and perhaps some day in the clinical practice. The ability to image active angiogenesis may enable earlier detection of vascular pathologies, and better evaluation of treatment options for patients with peripheral arterial disease. These could lead to more personalized therapeutc interventions and ultimately better patient outcomes.

## Methods

### Diabetic Animal Model

All experiments were completed with the approval of the Institutional Animal Care and Use Committee of the University of Illinois at Urbana-Champaign, following the principles outlined by the American Physiological Society on research animal use.

#### Glycated Serum Protein and Glycated Hemoglobin A1c

Male C57BL/6 mice (Jackson Laboratories) were used for all surgical and imaging interventions. A subset of mice (n = 11) underwent streptozotocin (STZ, Sigma-Aldrich, USA) treatment to induce type-1 diabetes mellitus (DM). STZ was administered via intraperitoneal injection at a dose of 40 mg/kg for 5 days. Glucosuria and fasting glycemia (>200 *mg*/*dL*) two weeks after the first STZ treatment marked the success of DM induction. Diabetic (6 weeks after STZ treatment, n = 11) and non-diabetic controls (n = 4) were anesthetized with 1–3% isofluorane vaporized in O_2_ at a rate of 1 L/min via nose cone. Glycated serum protein (GSP) represents a measure of short-to-medium term glucose control. GSP was measured colorimetrically in the serum of blood collected from DM (2 and 6 weeks after streptozotocin administration) and non-DM mice using an enzymatic assay (Diazyme, USA). Briefly, blood was collected in a covered Eppendorf tube from the jugular vein of the animal and allowed to clot by leaving it undisturbed at room temperature. Clots were removed by centrifugation at 1,000–2,000× g for 10 minutes in a refrigerated centrifuge. The resulting supernatant (serum) was used to measure GSP levels which were expressed in *μ* mol/L.

Glycated hemoglobin A1c (HbA1c) is an important indicator of long-term diabetic control. HbA1c was measured in whole blood collected from DM (at 2 and 6 weeks) and non-DM mice using a direct enzymatic assay (Diazyme, USA). The HbA1c concentration was expressed directly as a percent of total hemoglobin (THb) by use of a calibration curve.

#### Surgical Procedures

All animals underwent hindlimb occlusion of the right femoral artery and a sham operation on the left hindlimb, following previously-described procedures^15^. Briefly, a small incision was made on the right leg to expose the femoral vasculature, and dual ligation of the femoral artery was performed distal to the profundus branch to induce unilateral hindlimb ischemia. All mice were allowed to recover for 7 days after surgery.

#### Ischemia Validation

To validate the animal model, an additional group of normoglycemic mice (C57BL/6, n = 3), was used to verify the occlusion and the reduction of blood flow in ischemic hindlimb. Animals were imaged with laser doppler flowmetry Imager (moorLDI, Moor Instruments, UK) before, immediately after, and 1 week post-surgery. To confirm the presence of collateral vessels as a result of angiogenic and arteriogenic processes, Microfil (Flowtech Inc, USA) casting followed by the tissue clearing technique was performed on the same mice 4 weeks post-surgery using a procedure described previously^34^.

#### Biodistribution

One week after occlusion, 5 groups of animals were injected with 6.81 ± 0.536 MBq of ^64^Cu-NOTA-PEG4-cRGD_2_ via the jugular vein and at various time points (30 min n = 3, 1 hr n = 3, 2 hr n = 3, 4 hr n = 3, and 24 hr n = 2), different organs were excised and subjected to gamma well counting. The mass and radioactivity of tissue sections were assessed with a Voyager Pro. (Ohaus, USA) and Wizard2 gamma well counter (PerkinElmer, USA) respectively. The ^64^Cu signal was then corrected to account for background radioactivity, radioactive decay of the samples, and tissue weight.

### ***In Vitro*** Binding Specificity

To validate the specificity of FITC-PEG_4_-cRGD_2_ to the *α*_*V*_ *β*_3_ integrin receptor, human umbilical vein endothelial cells (HUVECs) were first cultured on coverslips until confluent, before being fixed using a 4% solution of paraformaldehyde for paired-staining with both FITC-PEG_4_-cRGD_2_ (approximately 1 *μ* M) and PE-labeled *α*_*V*_ *β*_3_ antibody LM609 (1:100, R&D Systems, USA). The cells on the coverslips were washed with buffer before and after each independent hour-long room temperature incubation, and the coverslips were ultimately mounted to microscope slides using DAPI Fluoromount (Southern Biotech). An Axiovert 200 M inverted fluorescence microscope (Zeiss, USA) was then used for imaging (employing the 10x and 20x objectives). Finally, the images were analyzed using the ZEN 2012 software package (Zeiss, USA).

Because diabetes is characterized by high blood glucose levels, which could potentially modify the structure of *α*_*V*_ *β*_3_ via glycosylation and alter ligand-receptor interactions, we performed additional experiments to assess cellular binding of PEG_4_-cRGD_2_ under high-glucose conditions. HUVECs were cultured in high-glucose (14 mM) growth media for 24 hours before incubation with FITC-PEG_4_-cRGD_2_ (0–1 *μ* M) at 4 °C for 2 hours. A control group was cultured in normoglycemic (5.5 mM) growth media followed by incubation with FITC-PEG_4_-cRGD_2_. Binding of FITC-PEG_4_-cRGD_2_ to *α*_*V*_ *β*_3_ integrin was assessed using flow cytometry (LSR II Flow Cytometry Analyzer, BD Biosciences, USA).

### ***In Vivo*** Imaging of PAD Associated Angiogenesis

One week after occlusion, DM and non-DM mice (n = 15) were injected with 6.81 ± 0.536 MBq of ^64^Cu-NOTA-PEG4-cRGD_2_ via the jugular vein and 60 minutes later *in vivo* microPET-CT imaging was performed using a small animal dedicated Inveon system (Siemens Healthcare USA). An additional group of non-diabetic mice (n = 4) was injected with 7.21 ± 2.31 MBq of ^64^Cu-acetate to study differential organ biodistribution of ^64^Cu-NOTA-PEG4-cRGD_2_. Animals were placed on a polyacrylic bed in the supine position with legs secured in an extended position. Mice underwent X-ray microCT imaging (80kVp, 500 *μ* A, 100 *μ* m spatial resolution) followed by 15 min microPET imaging (15% energy window centered at 511 keV). All mice were euthanized immediately after last imaging session was completed and tissue samples were taken for gamma well counting and snap-frozen in liquid nitrogen for immunofluorescence analysis.

#### Image Analysis

The microPET and microCT images were reconstructed using the OSEM/3D algorithm (Siemens Healthcare USA) and the cone-beam technique (Cobra Exim), respectively. MicroPET images were fused with microCT images and quantified using a semiautomated approach developed and evaluated previously^35^. Briefly, complex irregular volumes of interest (VOIs) were generated from the microCT images the proximal region was selected above the knee and near the ligation site where as the distal area was below the knee. The VOI’s were applied on the co-registered microPET images to calculate absolute ^64^Cu activities using the Inveon Research Workplace (Siemens Healthcare USA). These complex VOIs included only soft tissue (skeletal muscles) after the removal of bone structures during the image segmentation process. To validate the accuracy of the quantitative targeted imaging approach, image-derived results were compared with the gamma well counting analysis of the corresponding tissue samples.

### Postmortem Analysis

#### Gamma Well Counting

Skeletal muscles from both the ischemic and non-ischemic hindlimbs were excised and separated into distal and proximal sections based on the location of the ligature in the ischemic limb and anatomical landmarks in the non-ischemic limb. The mass and radioactivity of tissue sections were assessed with a Voyager Pro. (Ohaus, USA) and Wizard2 gamma well counter (PerkinElmer, USA) respectively. The ^64^Cu signal was then corrected to account for background radioactivity, radioactive decay of the samples, and tissue weight.

#### Histology and Immunofluorescence

Ischemic and non-ischemic hindlimb sections collected from diabetic and non-diabetic animals were embedded in the Optimal Cutting Temperature (OCT) compound. The embedded tissue sections were then snap frozen in liquid nitrogen and cut into 5 *μ* m sections using a cryotome. The cut sections were then fixed in ice-cold acetone and stained with either a Cy-5 fluorescent endothelial cell marker (CD31, EMD Millipore, USA), FITC-PEG_4_-cRGD_2_ (1 *μ* M), and inflammatory markers APC-CD14 (Santa Cruz Biology,USA), and PE-CD74 (R&D, USA). The stained sections were then relocated to an incubator and allowed to incubate overnight. After incubation, the stained sections were mounted using DAPI fluoromount (Southern Biotech, USA) and images were acquired using a fluorescent microscope (Zeiss Axiovert 200 M) at both 10× and 20× objectives. For processing, the acquired images were assessed using Zeiss Zen Blue software for the total area positively stained in several randomly chosen (200×) fields. This software was validated in a previous study conducted by our group^15^. Acquired stained images of the sections were assessed for co-localization between the different fluorescent antibodies (PE-CD74, APC-CD14, and Cy-5-CD13) and the FITC-PEG_4_-cRGD_2_. Different antibody channels were separated and quantified using an ImageJ-plugin (Coloc2) to obtain the Pearson’s correlation coefficient. This assessment was repeated for each of the images randomly chosen for percent positive area quantification.

### Statistical analysis

The Student t test was used to compare 2 groups DM vs non-DM. One-way ANOVA was used to compare multiple parameters. A value of *P* < 0.05 was considered significant.

## Conclusions

Diabetes is known to cause severe vascular complications that can lead to debilitating injury, and in some cases even death. While early detection of these complications remains a challenge, new methods involving focused regional PET-CT acquisitions of *α*_*V*_ *β*_3_ activity are showing promise as a means of assessing tissue angiogenesis *in vivo*. We have demonstrated this promise using a new molecular probe to image neovascularization within ischemic tissue in diabetic and non-diabetic mice. We also used a fluorescently-labeled analogue to evaluate process at the cellular-level. Our approach enables a quantitative spatio-temporal characterization of *α*_*V*_ *β*_3_ activity that may one day allow for the real-time evaluation of the therapeutic efficacies of different medical interventions (medicinal, surgical, or emerging genetic or cell-based interventions). The dimeric cRGD-based imaging probe displays intense focal retention, leading to *in vivo* acquisitions of superior image quality. Coupled with favorable blood clearance kinetics and optimal biodistributions, its potential for clinical use in imaging and monitoring angiogenesis in patients with peripheral vascular disease is significant.
